# Enhanced Delivery and Potency of Self-Amplifying mRNA Vaccines by Electroporation *in Situ*

**DOI:** 10.3390/vaccines1030367

**Published:** 2013-08-22

**Authors:** Yen Cu, Kate E. Broderick, Kaustuv Banerjee, Julie Hickman, Gillis Otten, Susan Barnett, Gleb Kichaev, Niranjan Y. Sardesai, Jeffrey B. Ulmer, Andrew Geall

**Affiliations:** 1 Novartis Vaccines & Diagnostics, Inc., 350 Massachusetts Ave, Cambridge, MA 02139, USA; 2 Inovio Pharmaceuticals, Blue Bell, PA 19422, USA

**Keywords:** antibodies, T cell responses, vaccine, HIV

## Abstract

Nucleic acid-based vaccines such as viral vectors, plasmid DNA (pDNA), and mRNA are being developed as a means to address limitations of both live-attenuated and subunit vaccines. DNA vaccines have been shown to be potent in a wide variety of animal species and several products are now licensed for commercial veterinary but not human use. Electroporation delivery technologies have been shown to improve the generation of T and B cell responses from synthetic DNA vaccines in many animal species and now in humans. However, parallel RNA approaches have lagged due to potential issues of potency and production. Many of the obstacles to mRNA vaccine development have recently been addressed, resulting in a revival in the use of non-amplifying and self-amplifying mRNA for vaccine and gene therapy applications. In this paper, we explore the utility of EP for the *in vivo* delivery of large, self-amplifying mRNA, as measured by reporter gene expression and immunogenicity of genes encoding HIV envelope protein. These studies demonstrated that EP delivery of self-amplifying mRNA elicited strong and broad immune responses in mice, which were comparable to those induced by EP delivery of pDNA.

## 1. Introduction

In 1990, Wolff *et al.* [1] demonstrated that direct injection of messenger RNA (mRNA) or plasmid DNA (pDNA) into the skeletal muscle of a mouse resulted in expression of the encoded protein. At the time, the feasibility of development of mRNA vaccines was considered uncertain because of instability *in vivo* and the technical difficulties in manufacturing RNA at large scale. Hence, much of the subsequent development of nucleic acid vaccines focused on pDNA. Older DNA vaccines have been shown to be immunogenic in a wide variety of animal species and several products are now licensed for commercial veterinary use. These include a West Nile virus vaccine for horses [2], an infectious hematopoietic necrosis virus vaccine for fish [3], a melanoma cancer vaccine for dogs [4], and a growth hormone releasing hormone gene therapy with electroporation delivery for pigs [5]. In humans, proof of principle for induction of both antibody and T cell responses by early pDNA vaccines has been demonstrated for various indications in several clinical trials [6,7,8]. However, the magnitude of these immune responses has been lower than those observed for conventional vaccines consisting of inactivated whole organisms or subunit proteins formulated with adjuvants. The reasons for the shortcomings of pDNA vaccines are not clear, but are likely due, at least in part, to inefficient delivery of pDNA into cells and inadequate stimulation of the immune system. The most promising approaches to overcome these limitations include facilitation of pDNA delivery by electroporation (EP) [9]; and stimulation of the immune system via the use of genetic adjuvants [10,11,12,13].

In parallel with the progress being made with pDNA, many of the obstacles to mRNA vaccine development have been addressed, resulting in a revival in the use of non-amplifying and self-amplifying mRNA for vaccine and gene therapy applications [14]. Naturally transient and cytosolically active mRNA is now seen by many [15] as a more viable potential vaccine platform. Direct injection of mRNA or “naked” delivery *in vivo* induces gene expression and generates immune responses [16,17,18,19], with self-amplifying mRNA being more efficient for gene expression *in situ* [17,20]. The potency of naked mRNA vaccines can be enhanced by cationic molecules [21], or lipid particles [17,22,23]. A recent study has demonstrated that formulated mRNA encoding influenza antigens is immunogenic and protective in animal models [24]. In addition, delivery of mRNA by the gene gun [25,26] or by EP at the site of injection [20,27,28] has been shown to improve immune potency. 

In this paper, we explore the utility of EP for the *in vivo* delivery of large, self-amplifying mRNA, as measured by reporter gene expression and immunogenicity of genes encoding HIV envelope protein. These self-amplifying mRNA vaccines differ from conventional mRNA in that they encode an RNA replicon from alphavirus engineered to efficiently undergo a single round of replication and amplify production of subgenomic mRNA encoding an antigen of interest [29]. Here we demonstrate that like EP delivery of pDNA encoding antigens (not replicons), EP delivery of self-amplifying mRNA elicited improved stronger and broader immune responses relative to non-EP delivered RNA. Both of these vaccine technologies have been previously shown to be effective in animal models and human clinical trials [6,18,19,30,31,32,33,34,35,36].

## 2. Results

### 2.1. Magnitude and Kinetics of Gene Expression *in Vivo*

**Figure 1:**
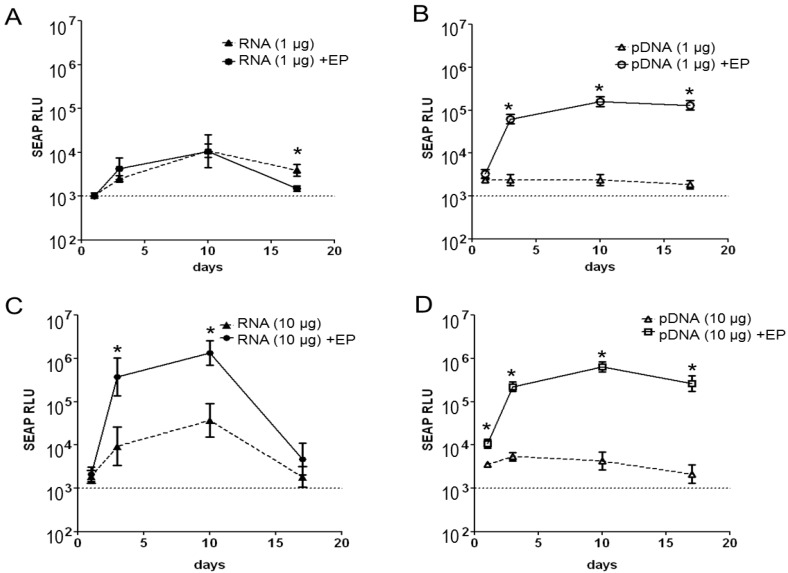
*In vivo* expression of secreted embryonic alkaline phosphatase (SEAP) after intramuscular njection of self-amplifying mRNA (RNA, 1 µg or 10 µg, panels **A**,**C**) or pDNA (DNA, 1 µg or 10 µg, panels **B**,**D**). The vectors were delivered by bilateral intramuscular injection with (+EP) or without electroporation. Serum SEAP expression was measured on days 1, 3, 10 and 17 after treatment, and represented as the log of mean relative luminescence (RLU)±SEM, n = 5/group. * Statistically significant (student-t test, *p* < 0.05).

After bilateral intramuscular injection of a low dose (1 μg) of naked self-amplifying mRNA encoding for the transgene secreted alkaline phosphatase (SEAP), measureable but low levels of serum SEAP were detectable as early as 3 days after treatment. No significant enhancements in the levels of SEAP expression were observed when the RNA was delivered using EP (RNA + EP) at the same dose, except at the latest time point (Figure 1A). In contrast to the RNA, mice injected with a low dose (1 μg) of naked pDNA showed levels of SEAP expression that were indistinguishable from baseline (Figure 1B), but substantial enhancements in SEAP expression was observed when pDNA was delivered by EP (pDNA + EP) at the same dose. At the 1 μg dose, SEAP expression by pDNA + EP was superior to RNA + EP.

After bilateral intramuscular injection of a higher dose (10 μg) of naked self-amplifying mRNA (Figure 1C), measureable levels of serum SEAP were detectable as early as 3 days after treatment and these levels were greater than those observed at the 1 μg dose (Figure 1A). Enhancements in the levels of SEAP expression were observed for RNA + EP by approximately 2-logs (days 3 and 10) compared to naked RNA delivery. High dose (10 μg) naked pDNA showed measurable but low levels of SEAP expression that were lower than those observed for the same dose of naked self-amplifying mRNA. Significant enhancements in SEAP expression were observed for pDNA + EP (Figure 1D). At the 10 μg dose, SEAP expression by RNA + EP was similar to pDNA + EP. Over the course of this 17-day study, it was observed that the kinetics of SEAP expression differed between self-amplifying mRNA (with and without EP) and pDNA. Mice injected with RNA showed measureable levels of expression at day 3, which peaked on day 10, and decreased towards background levels by day 17. On the other hand, mice administered pDNA delivered using EP, displayed high levels of SEAP expression at all time points tested, and these levels remained essentially undiminished for the duration of the study. 

### 2.2. Reporter Gene Expression *in Situ*

**Figure 2:**
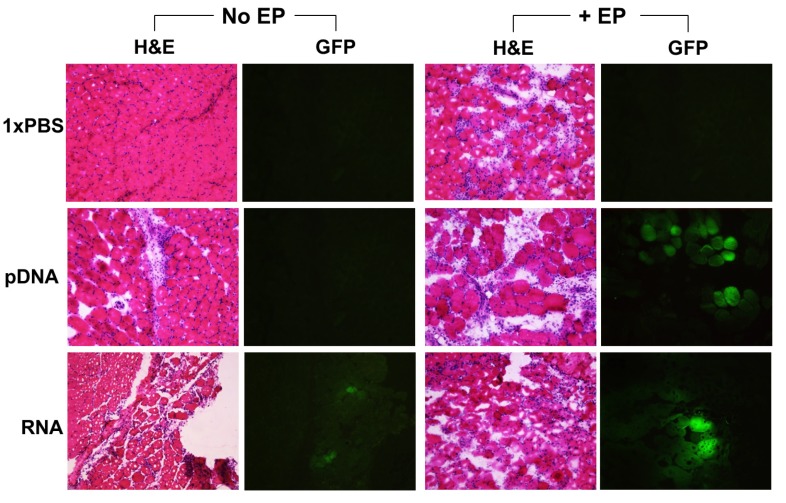
Histology of muscle tissues (transverse) by hematoxylin and eosin (H&E) staining, and expression of reporter protein (GFP) as delivered by intramuscular injection without EP (No EP) or with EP (+EP). Mice were injected intramuscularly with 50 µL of 1xPBS, pDNA or self-amplifying RNA vectors (pDNA or RNA, 5 µg/site). Images were obtained 2 days following treatment and are shown as representative of 6–8 slides taken per area, n = 4 muscles per group.

To assess the effect of EP-enhanced delivery of nucleic acid vaccines in muscle, mice were injected intramuscularly with pDNA or self-amplifying mRNA (5 μg/site) with or without EP. The treated muscles were excised for GFP expression analysis and for hematoxylin and eosin (H&E) staining. Representative images of GFP expression in mouse muscle are shown in Figure 2. Robust GFP expression was detected in both pDNA + EP and RNA + EP treated groups. Low levels of GFP expression were detected in the RNA (no EP) control group, but not in the pDNA (no EP) or 1× PBS control groups. These results are consistent with that observed in the SEAP studies.

Potential muscle damage related to pDNA and RNA treatment was assessed in mouse quadriceps 2 days after injection. Mouse muscle injected with 1× PBS only, was used as a treatment control. As shown in Figure 2, the H&E stain for this representative muscle demonstrates typical muscle physiology, with ordered, tightly bundled muscle fibers and some circulatory leukocyte activity. All groups receiving EP treatment demonstrated inflammation at the treatment site, as characterized by an increase in infiltration, consisting mainly of macrophages and neutrophils. There was also a breakdown in the ordered, bundled muscle fibers, typical of an inflammation response. Groups that received either pDNA or RNA (no EP) showed lower levels of leukocyte infiltration and no evidence of muscle fiber dissociation. Hence, injection of pDNA and RNA, with or without EP, induced local inflammation, but the combination of these nucleic acid vectors with EP demonstrated a higher inflammatory response. 

### 2.3. Immunogenicity of pDNA and Self-Amplifying mRNA Vaccines

**Figure 3:**
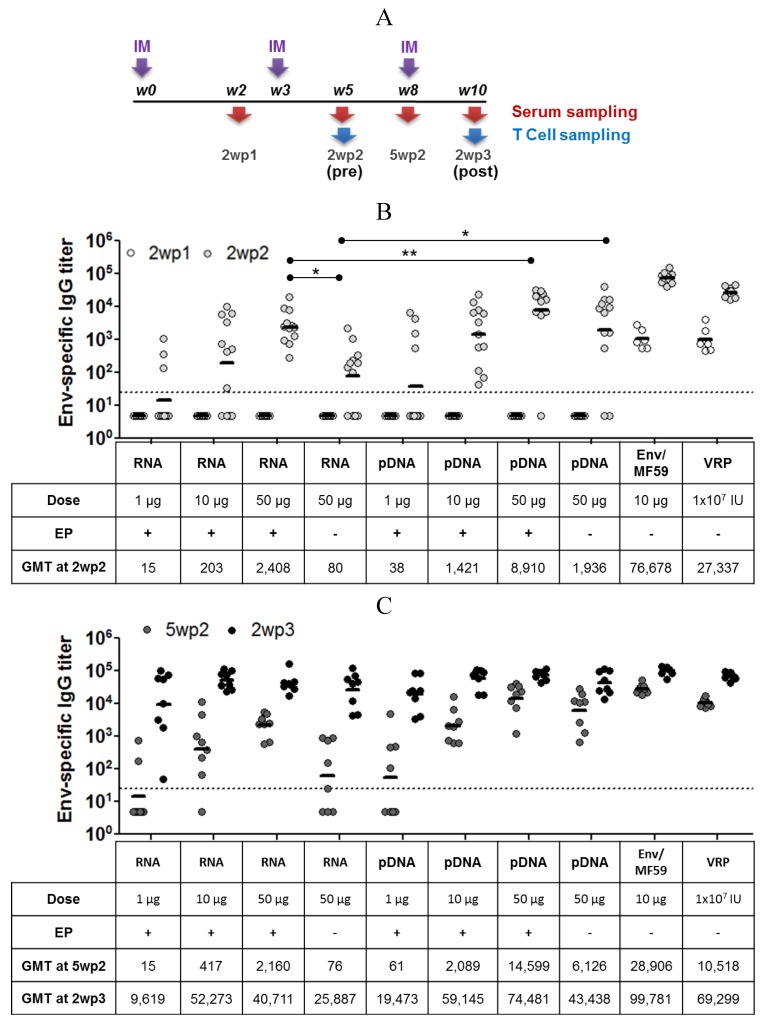
Study timeline for assessment of self-amplifying mRNA vector in an HIV immunogenicity model. (**A**) Female Balb/C mice received 2 treatments of RNA or pDNA vectors with or without EP (RNA or pDNA, 1, 10 and 50 µg), MF59-adjuvanted gp140.SF162 HIV envelope protein (Env/MF59, 10 µg) or viral replicon particles (VRP, 1 × 10^7^ IU) expressing the HIV Env-protein at weeks 0 and 3. All groups then received a single injection of 10 µg Env/MF59 at week 8. Serum and mucosal samples, and spleens for T-cell quantitation were collected on week 2 (2wp1), 5 (2wp2, pre-protein boost) 8 (5wp2) and 10 (2wp3, post-protein boost). Env-specific total IgG titer for all groups at 2wp1 and 2wp2 (**B**) and 5wp2 and 3wp2 (**C**) is plotted as log of mean titer, and the horizontal bar represents geometric mean titer (GMT). Titers < 25 (dotted line) were assigned a value of 5 for calculation of GMT. n = 6 at 2wp1, n = 12 at 2wp2, and n = 8 at 5wp2 and 2wp3. * denotes statistical significance with *p* < 0.05; ** denotes statistical significance with *p* < 0.005.

**Figure 4:**
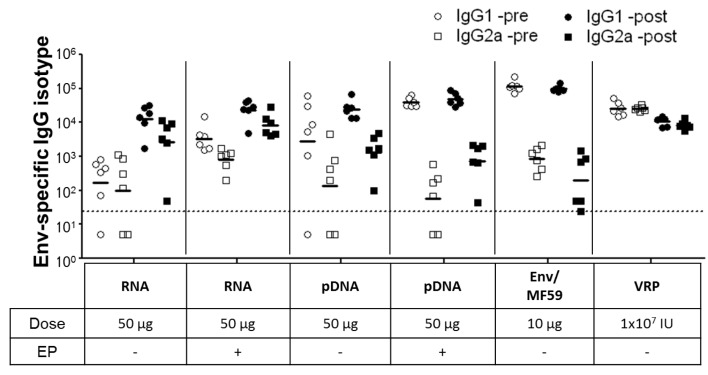
Env-specific IgG1 and IgG2a titers in mice sera were measured at 2wp2 (pre-) and 2wp3 (post-protein boost) for nucleic acid vectors with or without EP (RNA and pDNA; 50 µg), MF59-adjuvanted Env (Env/MF59, 10 µg) or viral replicon particles (VRP, 1 × 10^7^ IU) primed groups. Individual titers are plotted and the horizontal bar represents geometric mean titer (GMT) for each group (n = 6 per group). Titers <25 (dotted line) were assigned a value of 5 for calculation of GMT.

**Figure 5:**
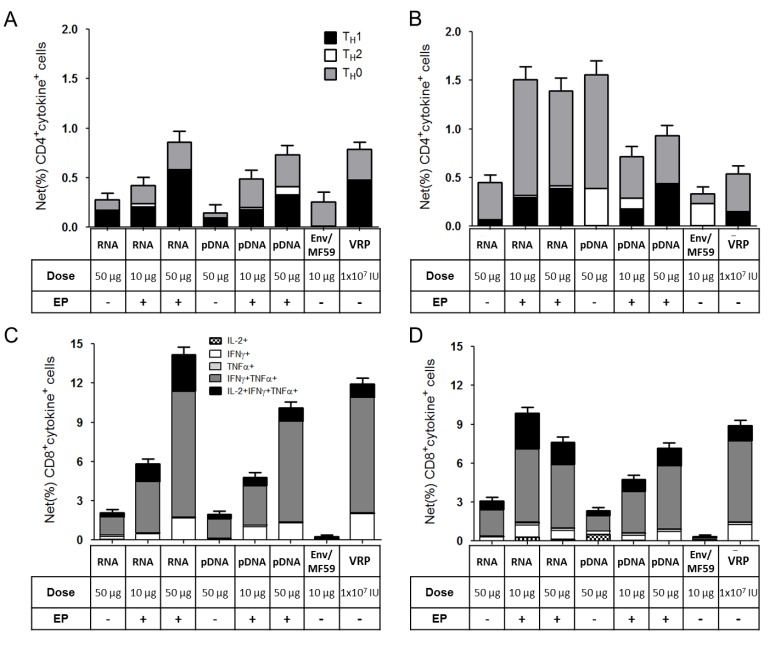
T-cell responses for groups treated with self-amplifying mRNA (RNA, 10 and 50 µg), pDNA (pDNA, 10 and 50 µg), MF59-adjuvanted Env (Env/MF59, 10 µg) or viral replicon particles (VRP, 1 × 10^7^ IU) at weeks 5 (**A**,**C**) and 10 (**B**,**D**). Pooled splenocytes (6 spleens/pool) were stimulated with HIV Env-derived antigenic peptides, stained for intra-cellular cytokines, and subjected to flow cytometry (methods). Graphs show the Env-specific (%) frequencies of CD4^+^ (**A**,**B**) and CD8^+^ (**C**,**D**) T-cells with error bars denoting 95% confidence limits.

Self-amplifying mRNA vectors expressing the HIV envelope protein gp140.SF162 (Env) were used to vaccinate mice using a heterologous prime-boost regimen (Figure 3A). Mice were administered 2 doses of self-amplifying mRNA, as the prime, and a third vaccination with recombinant HIV Env protein adjuvanted with MF59 (Env/MF59), as the boost. Viral replicon particles (VRP) and pDNA expressing the same antigen were used as benchmarks. Serum and spleens were collected at the specified time points (Figure 3A). Antibody responses were measured by ELISA for total IgG (Figure 3B,C) and IgG isotypes (Figure 4), and T cell responses were evaluated using an intracellular cytokine immunofluorescence assay (Figure 5). 

Env-specific IgG responses following RNA + EP or pDNA + EP treatments were only detectable after the 2nd immunization at all the doses tested (1, 10 and 50 µg, Figure 3B). Overall, the results show a dose response for both RNA and pDNA immunized groups, with pDNA producing higher Env-specific IgG titers at 2 weeks after the 2nd immunization (2wp2). At the high dose (50 μg), RNA+EP treatment was significantly more immunogenic than naked RNA (no EP) delivery, as measured by Env-specific IgG levels (GMT of 2,408 *vs.* GMT of 80), demonstrating the benefits of EP for the delivery for self-amplifying mRNA. EP delivery of pDNA also resulted in higher antigen-specific IgG levels (GMT of 8,910 *vs.* GMT of 1,936), but this increase was not statistically significant. Vaccination with VRP or Env/MF59 resulted in seroconversion of all the mice after a single dose and significantly boosted after the 2nd dose (Figure 3B). A reduction in titer of 2–3 fold was observed during the 3 weeks following (between 2wp2 and 5wp2) for these groups, which was not seen in case of groups primed with RNA and pDNA vaccines. The highest responding groups at 2 weeks after the 3rd immunization (2wp3) were those vaccinated three times with Env/MF59 (GMT of 99,781, Figure 3C). Immune responses for all groups that received 2 doses of nucleic acid vaccines (RNA, pDNA and VRP) were substantially boosted following administration of 10 µg of Env/MF59. Sera from mice immunized with 50 µg RNA or pDNA, 10 µg Env/MF59 or 1 × 10^7^ IU VRP were also assayed for antigen-specific IgG1 and IgG2a responses before (“-pre”, at 2wp2) or after protein boost (“-post”, at 2wp3). Groups primed with RNA (with or without EP) exhibited a balanced response pre- and post-protein boost, with IgG1 titers being slightly elevated compared to IgG2a (Figure 4). In contrast, the pDNA primed groups (with or without EP) and those that received Env/MF59 only produced a response significantly skewed towards IgG1 production over IgG2a, suggesting that RNA-based vaccines are more effective than plasmid DNA at stimulating Th1-type helper T cell responses.

Splenic Env-specific T-cell responses for both the RNA and pDNA vaccinated mice demonstrated a dose response, when assessed at the 2wp2 (pre-boost) time-point (Figure 5). The net frequencies of Env-specific CD4^+^ and CD8^+^ T-cells were ~4–5-fold higher for mice treated with 50 µg of RNA+EP compared to those that received RNA without EP. Similar enhancements were observed for pDNA, demonstrating the benefit of EP delivery for both types of nucleic acid vaccines on T-cell responses. The phenotypes of the T cell responses were predominated by Th0/Th1 for nucleic acid vaccines (RNA, pDNA and VRP) and Th0/Th2 for the protein vaccine (Figure 5A,C). Upon boosting with Env/MF59, this relationship was largely maintained. The protein vaccine alone elicited CD4^+^ T cell responses (Figure 5A), but not CD8^+^ T cell responses (Figure 5C). Hence, as might be expected, protein boosting of the nucleic acid-primed mice increased CD4^+^ T cell responses (Figure 5B), but not CD8^+^ T cell responses (Figure 5D). Overall, EP enhanced the potency of Th0/Th1 CD4^+^ and CD8^+^ T cell responses for the self-amplifying mRNA vaccine, as has been previously demonstrated for pDNA [37,38].

## 3. Discussion

This report demonstrates that EP is an effective means of increasing the delivery and potency of self-amplifying mRNA vaccines. These enhancements were similar in scope and magnitude to those seen for pDNA vaccines, despite several key differences between the two types of nucleic acid vaccines. First, pDNA vaccines require delivery into the cell nucleus for activity. Achieving a high level of antigen expression from pDNA vector is particularly challenging in the absence of EP in non-dividing cells such as mature myocytes. The delivery of pDNA past the nuclear barrier is considered a rate-limiting step in vaccine effectiveness, but can be overcome by EP. In contrast, RNA vaccines (both mRNA and self-amplifying mRNA replicons) are active in the cell cytoplasm, hence do not require delivery into the nucleus. Second, upon delivery into the cytoplasm, the self-amplifying mRNA vaccine produces many copies of itself and the subgenomic mRNA encoding the transgene. The enhancement of pDNA and RNA vaccine potency by EP, suggests that EP facilitates nucleic acid delivery across both the plasma and nuclear membranes. Third, self-amplifying mRNA produce relatively short-lived expression *in situ*, possibly due to induction of apoptosis of transfected cells, as occurs in alphavirus infected cells [16,17]. In contrast, pDNA can remain functional in the nucleus of transfected cells for prolonged periods of time, resulting in long-lived expression of reporter genes [34,39,40]. EP delivery of RNA and pDNA did not alter these kinetic profiles. Finally, pDNA and mRNA vaccines likely stimulate the innate immune system in very different ways. *E.coli*-derived pDNA is known to signal via TLR9, which is believed to play a role in DNA vaccine potency [41]. In contrast, viral RNA has the potential to interact with TLR3, TLR7, TLR8, as well as the intracellular helicases RIG-I and MDA5 [42,43]. Previously, we have shown that effective non-viral delivery of self-amplifying RNA vaccines can be achieved in mice with lipid nanoparticles (LNPs), at low doses of RNA (0.1–1.0 μg) [23]. This novel vaccine technology was found to elicit broad, potent and protective immune responses against RSV F protein that were comparable to a viral delivery technology. This is in contrast to what was observed for EP delivery of self-amplifying RNA in these studies, which required higher doses and two vaccinations to achieve seroconversion, suggesting that there is room for improvement via optimization of EP conditions for RNA. Enhanced delivery of the RNA vaccines by EP was not detrimental to gene expression or vaccine potency. Interestingly, RNA vaccination resulted in a balanced IgG1/IgG2a response, in contrast to pDNA vaccinated groups which exhibited a response skewed towards IgG1. The interaction of non-viral vectors with early and/or late innate immune signaling is very complex and is unique for each types of vector, as demonstrated by our study. A better understanding of the mechanism of action of these vaccines is warranted through further research. 

We have previously shown that pDNA encoding reporter genes can be successfully delivered into muscle in different animal models such as guinea pig, rabbit, sheep, and mouse by EP [35,44,45,46]. In addition, the delivery of siRNA was facilitated using dermally targeted EP with good tissue tolerability [47]. Others have reported on the EP delivery of self-amplifying mRNA and shown significant enhancement of antibody and T cell responses [20], as well as transient tissue damage [28]. Here, we assessed reporter GFP transgene expression levels and tissue damage in mouse muscle using the Elgen 1000 EP device. GFP expression was found to be robust in groups treated with self-amplifying mRNA and pDNA followed by EP, likely the result of enhanced nucleotide delivery and uptake facilitated by EP. Tissue inflammation was observed in all groups treated with EP, and this damage appeared to be more significant when EP was combined with nucleic acid. In the absence of nucleic acid, the EP-related damage was limited to the immediate vicinity of the electrodes, which is likely due to a localized effect on the muscle fibers caused by the electrical field strength of the EP procedure. The increased effect of EP delivery of nucleic acid may be due to the additional specific innate immune stimulation provided by *E. coli*-derived pDNA or viral RNA. Qualitative assessment of the treated muscles suggested that groups treated with pDNA + EP induced higher levels of infiltration than the RNA+EP group. The reason for this difference remains unclear and should be investigated further.

In summary, EP is an efficient non-viral delivery method for self-amplifying mRNA vaccines and is an effective means of increasing vaccine potency, as has been well documented for pDNA. Substantial enhancements of RNA gene expression *in situ*, antigen-specific antibody titers and T cell responses (CD4^+^ and CD8^+^) were observed. Nucleic acid vaccines (both pDNA and RNA) have several attributes that make them attractive alternatives to protein-based vaccines and viral vectors. Our results show that while some differences exist between RNA and pDNA vaccination, these vectors elicit a broad-based immune response profile including Th1 and CD8^+^ T cells (unlike protein-based vaccines) and do not induce interfering anti-vector immunity (unlike viral vectors). Enabling delivery technologies, such as EP, are critical to improve potency of nucleic acid vaccines in humans.

## 4. Materials and Methods

### 4.1. RNA Synthesis

The gene sequences for the SEAP (GeneBank accession #: U89937) and GFP (GeneBank accession #: KC896842.1) reporter genes and the HIV envelope protein antigen gp140.SF162 [48,49] were cloned into *Sal*I and *Xba*I sites of a modified VCR construct [29]. Plasmid DNA was purified by standard techniques and the nucleotide sequence of the inserts was confirmed by Sanger sequencing. pDNA was linearized immediately following the 3' end of the replicon by restriction digest and purified by phenol/chloroform extraction and ethanol precipitation. Linearized DNA template was transcribed into RNA using the MEGAscript T7 high-yield transcription kit (LifeTechnologies, Carlsbad, CA, USA). Transcripts were purified by LiCl precipitation, capped using the ScriptCap m^7^G Capping System (CellScript, Madison WI, USA) and the final product obtained by a second round of LiCl precipitation. RNA was then resuspended in PBS at the appropriate concentration prior to use, reporter or antigen gene expression was confirmed by Western blot analysis of transfected BHK cell lysates.

### 4.2. Plasmid DNA (pDNA) Preparation

For DNA vaccination, plasmids encoding the reporter gene SEAP or vaccine antigen (gp140.SF162 ) were constructed using standard molecular techniques using the previously described pCMVIII mamalian expression vector[49,50]. Plasmids were grown in *E.coli* and purified using Qiagen Plasmid Giga Kits (Qiagen, Valencia CA, USA). For histology experiments, gWiz-GFP plasmid DNA (Aldevron, Fargo ND, USA) was used.

### 4.3. Production of Viral Replicon Particles (VRPs)

To compare RNA vaccines to traditional RNA-vectored approaches for achieving *in vivo* expression of reporter genes or antigens, we used VRPs produced in BHK cells by previously described methods [29]. In this system, the antigen-expressing (or reporter gene-expressing) replicons consisted of alphavirus chimeric replicons (VCR) derived from the genome of Venezuelan equine encephalitis virus (VEEV) engineered to contain the 3' terminal sequences (3' UTR) of Sindbis virus and a Sindbis virus packaging signal (PS) (see Figure 2 of Perri *et al*.) [29]. These replicons were packaged into VRPs by co-electroporating them into baby hamster kidney (BHK) cells together with defective helper RNAs encoding the Sindbis virus capsid and glycoprotein genes (see Figure 2 of Perri *et al*.). The VRPs were then harvested and titrated by standard methods and inoculated into animals as solutions in PBS.

### 4.4. MF59-Adjuvanted gp140.SF162 HIV Envelope Protein Subunit Vaccine Candidate

The subunit vaccine was expressed, purified and formulated as previously described [48,49].

### 4.5. *In Vivo* Immunization

Female Balb/c mice at 6–8 weeks old (Charles River Laboratories, Worcester, MA, USA) were housed and handled according to the standards of the Institutional Animals Care and Use Committee. Electroporation was carried out by the Elgen DNA Delivery System (Inovio, San Diego, CA, USA). Prior to immunization, the mice were anaesthetized with isofluorane. A 30 µL volume of DNA or RNA was injected into the quadricep muscle of the hindleg, followed by insertion of 2-needle electrode that flanks the injection site. Two pulses of electricity at 60 ms duration/pulse and 80 V was delivered to the quadriceps muscle. This procedure was repeated on the second hindleg. A total dose of 1 or 10 µg DNA or RNA were delivered per animal.

For the histology experiments, 3 week old Balb/c mice were purchased from Charles River (Wilmington, MA, USA) and housed at BioTox Inc (San Diego, CA, USA). Intramuscular injections were performed on mice quadriceps muscles using a 29 gauge syringe delivering either 1× PBS, 5 µg gWiz-GFP plasmid DNA (Aldevron, Fargo, ND, USA) or 5 µg GFP-expressing self-replicating RNA vector (Novartis, Cambridge, UK). The injection volume was maintained at 30 µL across all groups. Immediately following IM injection, EP was performed by inserting the 2-needle array (27-gauge with 4 mm spacing) of the Elgen 1000 EP device (Inovio Pharmaceuticals, San Diego, CA, USA) into the mouse muscle. The parameters for the EP were 2, 80 V pulses of 60 ms same as described above. Mice were humanely sacrificed by cervical dislocation 48 hours post-treatment and muscles were excised post mortem.

For immunogenicity experiments, Balb/c mice at 6–8 weeks old were injected with 30 µL of pDNA or RNA at respective doses in the quadriceps muscles of the hindleg and followed by electroporation where required with 2 pulses of 100 V at 60 ms duration. VRP and Env/MF59 were delivered by intramuscular injection to the quadriceps at 50 µL per leg to both legs. A total dose of 1 × 10^7^ IU or 10 µg was delivered per animal, respectively.

### 4.6. Histology

To assess GFP expression in mouse muscle, excised quadriceps were embedded in OCT (Sakura-Finetek, Torrance, CA, USA) and snap-frozen in liquid nitrogen (LN2)-cooled isopentane (Sigma-Aldrich, St. Louis, MO, USA). The fresh-frozen samples were stored at −80 °C until the cryosectioning procedure. The cryosectioning was performed on a Bright Model OTF cryostat machine. To sample the muscle tissue, two serial 15 µm cross-sections were taken and adhered to separate gelatin-coated slides. Separate slides were prepared for fluorescence imaging and for H&E staining. A 15 µm section was taken every 75 µm through the muscle. Thirty-six sections were prepared for each muscle. Once cryo-sectioned, the tissues were fixed in 4% paraformaldehyde/1× PBS solution for 10 minutes and then washed 3 × 5 minutes with 1× PBS. The H&E staining procedure was carried out using Histo·Perfect™ H&E Staining Kit (BBC Biochemical, Seattle, WA, USA) following the manufacturer’s instructions Briefly, tissues were rehydrated using increasingly dilute ethanol (100%–70%) and DI water and then stained with hematoxylin for five minutes. Slides were rinsed with DI water and submerged into proprietary Acid Wash and Blueing Solutions. The tissues were stained with Eosin for 45 seconds and then washed with proprietary S2 Histo Wash. Finally, slides were dehydrated in xylene for 1 minutes and cover-slipped using xylene-based mounting media. Representative depictions of fluorescence and bright field images were taken using an Olympus BX51 microscope (Olympus of Americas, Center Valley, PA, USA) and MagnaFire SP S camera system (Olympus of Americas, Center Valley, PA, USA) at a 10× magnification. The microscope and camera settings remained constant across all groups.

### 4.7. Quantitation of SEAP Expression

The serum from all groups and time points was sampled for SEAP expression using the *Phospha-Light* Chemiluminescence Assay Kit (Applied Biosystems, Foster City, CA, USA). The assay was performed according to manufacturer’s protocol for a 96-well plate format, using Microlite* Luminescence Microtiter plates (Thermo Fisher, Waltham, MA, USA). The SEAP chemiluminescence was detected in a luminometer (Berthold Technologies) using 1 seconds duration for signal integration.

### 4.8. ELISA for Antigen-Specific Antibodies

Detection of gp140.SF162 specific antibodies in serum samples was performed by ELISA using plates coated with 100 µg gp140.SF162 per well, in 96-well flat bottom polystyrene plate (Nunc MaxiSorp, Thermo Fisher, Waltham, MA, USA). The plates were incubated SuperBlock blocking buffer in 1× PBS (Pierce) for 2 hours at 37 °C and then exposed to mouse serum diluted 5-fold serially in assay diluents (5% Goat Serum and 0.1% Tween 20 in 1× PBS). The plates were incubated at 37 °C for 2 hours and washed 3 times with wash buffer at 200 µL/well (1× PBS and 0.1% Tween 20). HRP-conjugated goat anti-mouse IgG, IgG1 or IgG2a (Southern Biotech, Birmingham, AL, USA) prepared in assay diluent was added to the plates at 100 µL/well, followed by 1 hour incubation at 37 °C. The plates were washed 3× with wash buffer, and the amount of HRP bound was quantified using TMB detection system (KPL Labs). Briefly, 100 µL of prepared TMB reagent was added to each well. The plate was immediately transferred to a dark environment for 15 minutes. At the end of this incubation, 1 M H_3_PO_4_ was added at 100 µL/well, and chromatic absorbance at OD 450 nm was recorded for each well. Each sample was assayed in duplicate and the average absorbance was used for titer analysis. A standard made of mouse serum with known response to gp140.SF162 was also included in each plate to normalize responses among the plates. The titer each sample was reported as the reciprocal of fold dilution at a pre-determined OD 450 nm, which was 20% of the absorbance of the standard at the highest dilution.

### 4.9. Intracellular Cytokines Immunofluorescence Assay

Four spleens from identically vaccinated BALB/c mice were pooled and single cell suspensions were prepared. Two antigen-stimulated cultures and two unstimulated cultures were established for each splenocyte pool. Cultures contained 1 × 10^6^ splenocytes, anti-CD28 mAb (BD Pharmingen, #553294; 1 μg/mL), and Brefeldin A (BD Pharmingen, #555029, 1:1,000). HIV-1 SF162 Env-specific T cells were stimulated with a pool of Ia^d^-restricted 20mers (YGVPVWKEATTTLFCASDAK, AYDTEVHNVWATHACVPTDP, ITQACPKVSFEPIPIHYCAP, NVSTVQCTHGIRPVVSTQLL) and H-2D^d^ restricted 9mer (IGPGRAFYA), each at a final concentration of 2.5 µg/mL. Unstimulated cultures did not contain peptides, and were otherwise identical to the stimulated cultures. After culturing for 6 hours at 37 °C, cells were washed and then stained with Pacific Blue labeled anti-CD4 (BD Pharmingen, #558107) and Alexafluor 700anti-CD8 (BD Pharmingen, #557959) monoclonal antibodies (mAb). Cells were washed again and then fixed with Cytofix/cytoperm (BD Pharmingen, #554714) for 20 minutes. The fixed cells were then washed with Perm-wash buffer (BD Pharmingen, #554714) and then stained with a cocktail of PerCP-Cy5.5-labeled anti-IFN-γ (Ebiosciences, #45-7311-80), Alexafluor 488-labeled anti-TNF-α (BD Pharmingen, #557719), Allophycocyanin-labeled anti-IL-2 (BD Pharmingen, #554429), and Phycoerythin-labeled antiIL-5 (BD Pharmingen, #554395). Cells were washed and then analyzed on an LSR II flow cytometer (BD Pharmingen). FlowJo software (Tree Star, Inc., Ashland, OR, USA) was used to analyze the acquired data. The CD4^+^8^−^ and CD8^+^4^−^ T cell subsets were analyzed separately. For each subset in a given sample the % cytokine-positive cells was determined. The net (%) antigen-specific T cells were calculated as the difference between the % cytokine-positive cells in the antigen-stimulated cultures and the % cytokine-positive cells in the unstimulated cultures. The contribution of the various T_H_ subsets to the overall CD4^+^ T-cell response was plotted using these criteria: T_H_1: CD4^+^IFNγ^+^IL-5^neg^, T_H_2: CD4^+^IFNγ^neg^IL-5^+^, T_H_0: CD4^+^IL-2^+^/TNFα^+^and IFNγ^neg^IL-5^neg^. The 95% confidence limits for the % antigen-specific cells were determined using standard methods [50].

### 4.10. Statistical Analysis and Graphing

All statistical analyses were performed using GraphPad Prism 5 software (GraphPad Software, La Jolla, CA, USA). For analysis of SEAP expression, student-t test was used with confidence limit of *p* < 0.05. 

## 5. Conclusions

Self-amplifying RNA vaccines hold promise as next generation nucleic acid vaccines, but may require delivery technologies to enhance potency. Electroporation in situ has been demonstrated to be very efficient for increasing delivery and effectiveness of plasmid DNA vaccines. We show here that electroporation can also be used to markedly enhance the delivery and potency of RNA-based vaccines.
